# Molecular Mechanisms of Isolated Polycystic Liver Diseases

**DOI:** 10.3389/fgene.2022.846877

**Published:** 2022-04-26

**Authors:** Ziqi Yu, Xiang Shen, Chong Hu, Jun Zeng, Aiyao Wang, Jianyong Chen

**Affiliations:** ^1^ Munich Medical Research School, LMU Munich, Munich, Germany; ^2^ Department of Gastroenterology and Hepatology, The First Affiliated Hospital of Nanchang Medical College, Jiangxi Provincial People’s Hospital, Nanchang, China

**Keywords:** polycystic liver disease, PLD, PCLD, PRKCSH, Sec63, LRP5, alg8

## Abstract

Polycystic liver disease (PLD) is a rare autosomal dominant disorder including two genetically and clinically distinct forms: autosomal dominant polycystic kidney disease (ADPKD) and isolated polycystic liver disease (PCLD). The main manifestation of ADPKD is kidney cysts, while PCLD has predominantly liver presentations with mild or absent kidney cysts. Over the past decade, *PRKCSH*, *SEC63*, *ALG8*, and *LRP5* have been candidate genes of PCLD. Recently, more candidate genes such as *GANAB*, *SEC61B*, and *ALR9* were also reported in PCLD patients. This review focused on all candidate genes of PCLD, including the newly established novel candidate genes. In addition, we also discussed some other genes which might also contribute to the disease.

## Introduction

### PLD and PCLD

Polycystic liver disease (PLD) is a rare autosomal dominant disorder characterized by multiple fluid-filled cysts in the liver (Russell and Pinson, 2007; Boerrigter et al., 2021). It is normally diagnosed by CT scans, ultrasound, and magnetic resonance imaging (Qian, 2010). Most of the PLD patients (>80%) are asymptomatic with preserved synthetic function and parenchymal liver volume (Qian, 2010; Gevers and Drenth, 2013). However, due to hepatomegaly, some patients might present abdominal pain, nausea, dyspnoea, and early satiety (Everson and Taylor, 2005). Occasionally, hemorrhage or infection in cysts due to cystic rupture could cause severe abdominal pain, fever, and leukocytosis (Russell and Pinson, 2007). The risk factor of PLD includes gender (with the majority of female patients), usage of estrogens, and multiple pregnancies (Sherstha et al., 1997; Alvaro et al., 2006; Gevers and Drenth, 2013). Normally there are no specific laboratory abnormalities in patients with PLD. Only gamma-glutamyl transferase (gGT) was increased in 51% and alkaline phosphatase (AP) was elevated in 17% of the patients (Van Keimpema et al., 2011).

Surgical treatment of PLD includes aspiration and sclerotherapy, fenestration, hepatic resection, and liver transplantation. However, all therapies except for liver transplantation have risks of recurrence, and only liver transplantation is the curative way (Drenth et al., 2010). Drug therapy such as somatostatin analogs and mTOR inhibitors are normally used in treating PLD. Somatostatin analogs such as octreotide and lanreotide could significantly reduce approximately 4% the liver volumes after 1 year of treatment. The cessation of treatment would cause a recurrence of liver growth (Chrispijn et al., 2012). A single case series using mTOR inhibitor sirolimus showed a significant reduction in liver volume (Qian et al., 2008). However, other clinical randomized studies of mTOR inhibitors such as everolimus and sirolimus had no significant effect on cystogenesis (Serra et al., 2010; Walz et al., 2010; Chrispijn et al., 2013).

Genetically, PLD consists of two different types: isolated polycystic liver disease (PCLD) and PLD with autosomal dominant polycystic kidney disease (ADPKD). ADPKD, including PKD1 (OMIM #173900) and PKD2 (OMIM #613095), is the main hereditary kidney disease that causes chronic renal failure. More than half of the patients with ADPKD would present liver cysts in the later stage (Hoevenaren et al., 2008). However, PCLD is normally without or only presents mild kidney cysts, and the prognosis of PCLD is normally better than ADPKD (Chandok, 2012). In OMIM, four types of PCLD are included: PCLD1 (OMIM #174050) caused by heterozygous mutation in *PRKCSH*, PCLD2 (OMIM #617004) caused by heterozygous variant in *SEC63*, PCLD3 (OMIM #617874) caused by heterozygous mutant *ALG8*, and PCLD4 (OMIM#617875) caused by heterozygous mutation in *LRP5*. Among these four types, PCLD1 and PCLD2 are the most common types of PCLD. Mutations in *PRKCSH* and *SEC63* could explain 20%–41% of the cases (Van Keimpema et al., 2011; Gevers and Drenth, 2013; Masyuk et al., 2018). ADPKD and PCLD are both autosomal dominant diseases, which means most of the patients may carry a *de novo* mutation or have a positive family history. For people with a positive PCLD family history, genetic testing is suggested, such as whole-exome sequencing as a precaution. It is also beneficial for the diagnosis, surveillance, and regulation of diseases. For patients already diagnosed with PCLD, genetic counseling is also recommended.

Moreover, Janssen et al. (2011) found that 76% of the cyst from patients carrying a germline heterozygous *PRKCSH* mutation acquired a somatic second mutation in the wild-type allele. The germline mutation serves as the “first hit” and the somatic as the “second hit,” resulting in the loss of heterozygosity (LOH) of *PRKCSH*, which might be the reason for cyst formation. Meanwhile, Janssen et al. (2012) also found that the LOH in *SEC63* in patients with germline *SEC63* mutations confirmed the possibility of the “second hit” in cytogenesis. This explanation could also explain the high variability in disease presentations in PCLD patients (van Aerts et al., 2018).

### Genetics

#### PRKCSH

**FIGURE 1:**
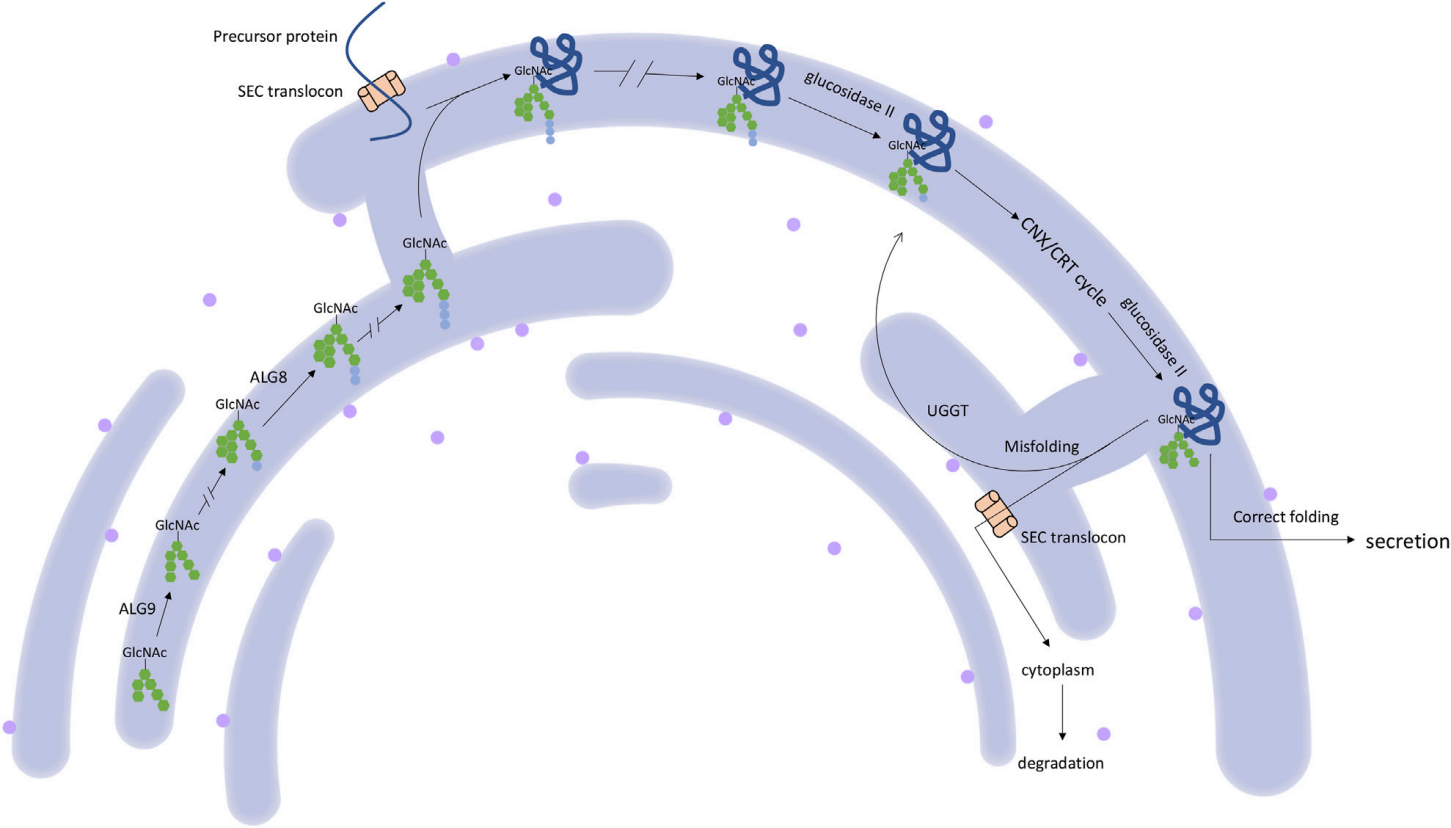
The schematic of the role of the PCLD genes in the protein synthesis process in ER.

**FIGURE 2:**
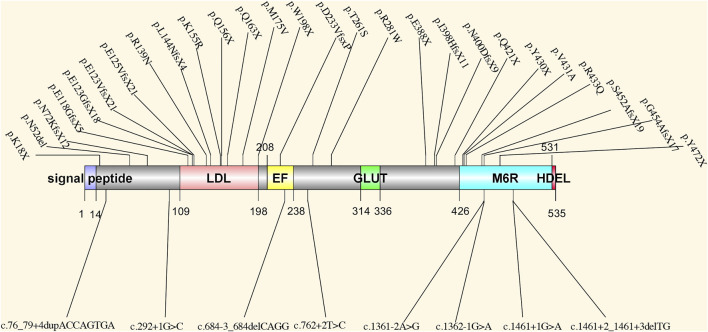
The spectrum of all reported mutations in *PRKCSH*. LDL: low-density lipoprotein class A domain; EF: EF-hand domain; GLUT: a glutamic acid-rich segment; M6R: mannose-6-phosphate receptor-binding domain; HDEL: ER retrieval signal His-Asp-Glu-Leu tetrapeptide.

*PRKCSH* encodes protein kinase C substrate 80K-H, also called the beta-subunit of glucosidase II (Li et al., 2003), which is a highly conserved protein. This protein contains a signal peptide domain, a low-density lipoprotein class A (LDL) domain, an EF-hand domain, a glutamic acid-rich (GLUT) segment, a mannose-6-phosphate receptor-binding domain (M6R), and an ER retrieval signal His-Asp-Glu-Leu (HDEL) tetrapeptide. The noncatalytic beta-subunit can interact with the catalytic alpha-subunit of glucosidase II and facilitate the removal of 1,3-linked glucose from newly synthesized glycoprotein (Gao et al., 2010) (Figure 1). This process is essential for the folding and quality control of glycoprotein (Qian, 2010; Waanders et al., 2006). The linkage between *PRKCSH* and PCLD was established in 2000 and 2003 (Li et al., 2003; Reynolds et al., 2000). After that, more PCLD patients with mutations in *PRKCSH* were found. All reported mutations according to HGMD and ClinVar databases are summarized in Figure 2. According to the spectrum of mutations, we found that most of the mutations clustered in the LDL domain (11 out of 35 mutations, 31.4%) and the M6R domain (10 out of 35 mutations, 28.6%), while no mutation was found in the GLUT and the HDEL region. In regard to mutation types, 31.4% of the mutations are frameshift (11 out of 35), followed by splice (8 out of 35) and nonsense (8 out of 35) mutations, both accounting for 22.9% of the mutations. Loss-of-functional mutations, including nonsense, frameshift, and splice mutations, are 77.1% of the reported mutations. In the reports, the patients carrying mutant *PRKCSH* normally have mild PCLD with a positive family history or severe PCLD without a family history. PKD is normally absent or very mildly presented in the patients (Drenth et al., 2003; Li et al., 2003; Drenth et al., 2004; Peces et al., 2005; Waanders et al., 2006; Waanders et al., 2010; Besse et al., 2017). However, these studies were normally performed on patients who did not meet the clinical criteria of ADPKD. An overall genetic screening in PLD patients, including ADPKD and PCLD, could be performed to have a better view of the genetic distinction between ADPKD and PCLD.

#### SEC63

**FIGURE 3:**
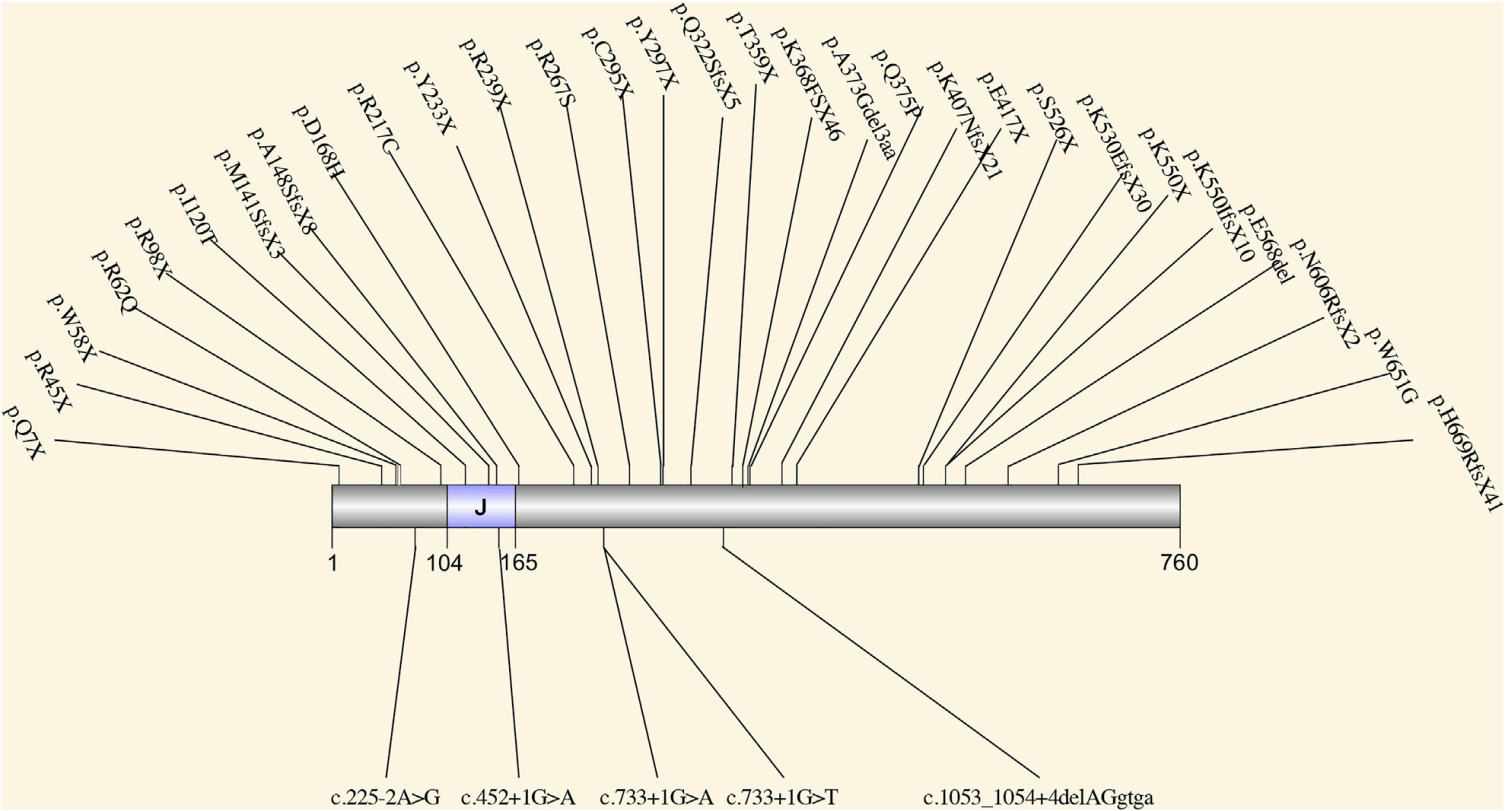
The spectrum of all reported mutations in *SEC63*. It contains a J-domain that can interact with the Hsp70 family of ATPase.

*SEC63* encodes an endoplasmic reticulum (ER) protein. This protein contains a J-domain that can interact with the Hsp70 family of ATPase (Misselwitz et al., 1999). SEC63, together with SEC61 and SEC62, comprises a translocon (Qian, 2010), which plays a role in precursor proteins translocation in ER (Waanders et al., 2006). This translocational process is upstream of the PRKCSH protein quality control process (Schnell and Hebert, 2003) (Figure 1). The translocon complex is also thought to play a role in the Ca^2+^ channel (Lang et al., 2017). The relationship between *SEC63* mutation and PCLD was first found in 2004. Seven loss-of-function mutations were identified in eight families with PCLD but without *PRKCSH* mutations (Davila et al., 2004). After that, more *SEC63* mutations were detected in PCLD patients. All reported mutations collected by HGMD and ClinVar database are summarized in Figure 3. These mutations are evenly distributed in all gene parts with no hotspot and cluster region. By considering the mutation types, nonsense mutation with 36.1% (13 out of 36) is the most common type, followed by frameshift mutation with 25% (9 out of 36). More missense mutations were reported in SEC63 with 19.4% (7 out of 36), which is different from the mutation types of *PRKCSH*. However, loss-of-function mutations, including nonsense, frameshift, and splice, are still the mainstream with 75%. In these studies, most of the patients recruited in screening for *SEC63* mutations were also PCLD patients with a positive family history or patients with severe and symptomatic PCLD (Davila et al., 2004; Waanders et al., 2006; Waanders et al., 2010; Kleffmann et al., 2012; Wills et al., 2016; Besse et al., 2017). Similar to patients with *PRKCSH* mutations, these patients also normally have no or very mild PKD.

#### ALG8

*ALG8* encodes a glycosyltransferase (Oriol et al., 2002) in ER that catalyzes the assembly of the second glucose residue to the lipid-linked oligosaccharide precursor (Aebi, 2013). It is a protein that plays a role in glycoprotein quality control in ER (van de Laarschot and Drenth, 2018). In 2017, (Besse et al., 2017) screened three heterozygous mutations (p. R364X, p. R179X, c.1038+1G>T) in the *ALG8* gene in five patients with PCLD. Four of the five patients present with severe PCLD with very mild PKD, and one patient presents moderate liver cysts without kidney assay. In their study, they genetically engineered knock-out of *Alg8* in mouse epithelial cell line and observed decreased levels of Pkd1 protein, defective posttranslational glycosylation, modification, and impaired trafficking of Pkd1 protein. These defects can be rescued by re-expression of wild-type *Alg8* cDNA in the *Alg8*−/− cells (Besse et al., 2017).

#### LRP5

*LRP5* is a single-pass transmembrane co-receptor for the canonical Wnt signaling (Sawakami et al., 2006). Wnt signaling cascade is required in many distinct developmental processes such as planar cell polarity, cell proliferation, fate decision, and stem/progenitor compartments regulation (Croce and McClay, 2008; Badders et al., 2009). The canonical Wnt signaling cascade is that after ligand binding to the Frizzled (Fzd) and the LRP5/6/arrow co-receptor complex, the cytoplasmic tail of co-receptor phosphorylated, leading to the recruitment of Axin and inhibition of GSK3β. This results in the inhibition of the phosphorylation of β-catenin and the dissociation of the β-catenin destruction complex, thus increasing the stability of β-catenin (Mao et al., 2001; Johnson et al., 2004; Nusse and Clevers, 2017; Lee-Law et al., 2019; Ren et al., 2021). Wnt proteins can also activate the Rho family (Wu et al., 2008), Ca^2+^ pathway (Kühl et al., 2000), PKCδ (Tu et al., 2007), and mTORC1 (Inoki et al., 2006), which consists of mTOR. mTOR was thought to play a role in cystogenesis (Chandok, 2012). Besides, a study also showed that PKD1 might be associated with Wnt signaling (Kim et al., 2016), indicating a possible linkage between PKD1 and LRP5.

The association between *LRP5* and PCLD was first established in 2014 (Cnossen et al., 2014). This study screened four heterozygous mutations (i.e., p.V454M, p.R1188W, p.R1529S, and p.D1551N in *LRP5*). All the patients carrying LRP5 mutations are symptomatic PCLD patients. All four mutations led to a decreased Wnt signaling. Patients with mutations p.R1188W and p.R1529S present with moderate to severe PCLD without renal cysts, while patients carrying p.D1551N have PCLD with three renal cysts and one patient carrying p.V454M has mild PCLD with several bilateral renal cysts. However, no significant differences could be found in the disease characteristics between PCLD patients with or without LRP5 mutation (Cnossen et al., 2014).

#### GANAB

**FIGURE 4:**
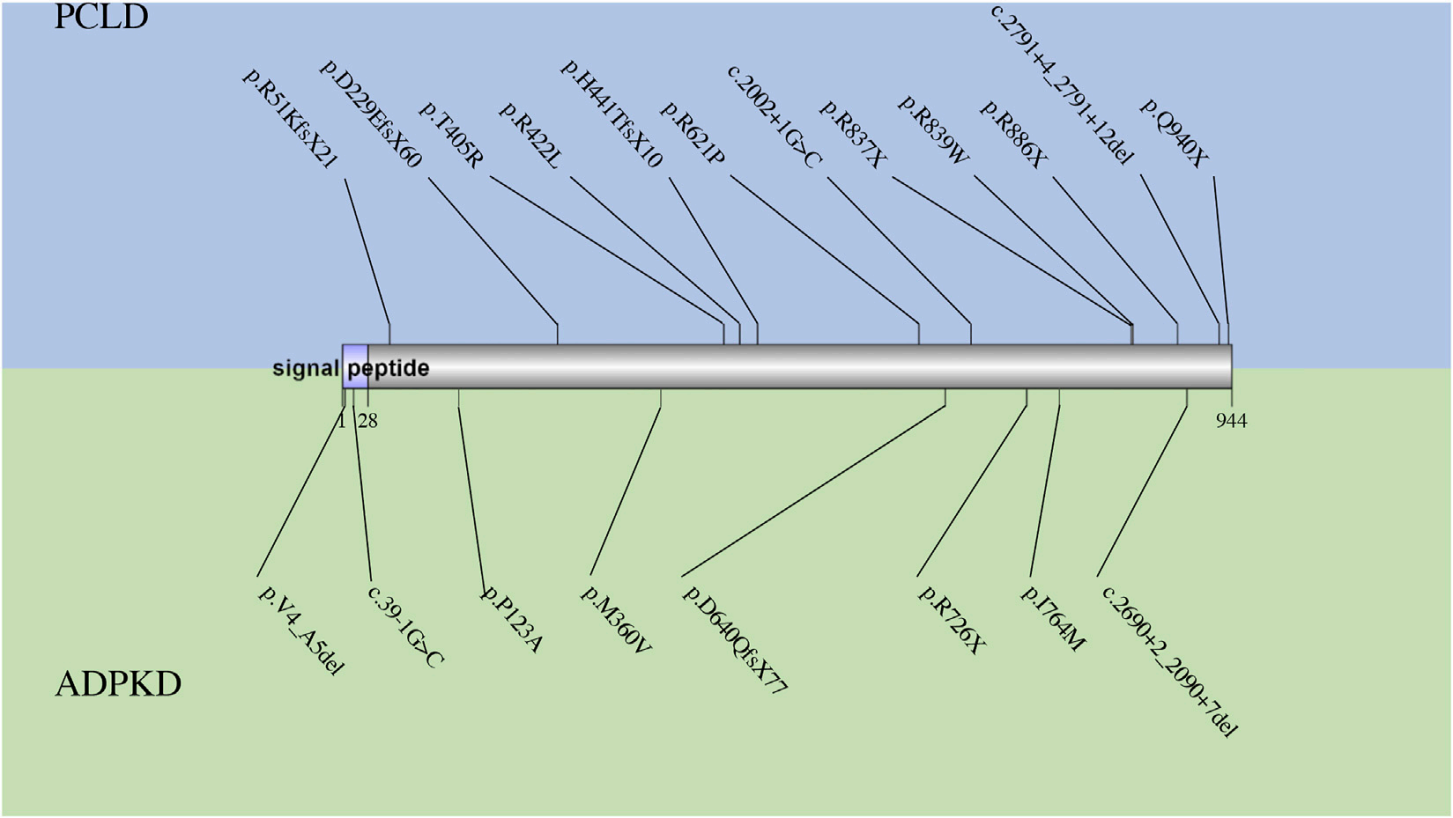
The spectrum of all reported mutations in *GANAB*. The mutations reported in PCLD are shown in blue, while those reported in ADPKD are shown in green.

*GANAB* encodes the catalytic alpha-subunit of glucosidase II in the ER lumen, which interacts with the noncatalytic beta-subunit encoded by *PRKCSH* and removes the glucose from glycoprotein (Gao et al., 2010; Porath et al., 2016) (Figure 1). Loss of function in *GANAB* would cause decreased expression level of PKD1 (Besse et al., 2017), reduced maturation and localization of PKD1 and PKD2 protein (Porath et al., 2016), and impaired trafficking of PKD1 past the Golgi apparatus (Besse et al., 2017). Although PCLD caused by the deficiency in *GANAB* was not included in OMIM, several dependent groups have successfully screened variants in *GANAB* in patients with PCLD or ADPKD. All mutations in the HGMD database are summarized in Figure 4. In patients diagnosed as PCLD carrying *GANAB* mutation, mild-to-severe PKD with absent to moderate PKD could be observed (Porath et al., 2016; Besse et al., 2017; Besse et al., 2018; van de Laarschot et al., 2020). Compared to the PCLD patients with other gene mutations, a higher frequency of PKD could be observed in these patients. This might be because *GANAB* is also a causative gene of PKD, and PKD patients sometimes also have PLD presentations.

#### SEC61B

Recently, *SEC61B* has been candidate gene in PCLD. This gene encodes the beta-subunit of SEC61 protein, which, together with SEC62 and SEC63, comprises the SEC61 translocon (Qian, 2010) (Figure 1). SEC61 has three subunits: alpha, beta, and gamma. Alpha-subunit consists of the SEC61 translocon channel pores, while gamma-subunit directly combines with alpha-subunit. However, the function of the beta-subunits is still under characterization (Lee-Law et al., 2019). However, research shows that the inactivation of SEC61B could result in a deprived PKD1 expression level (Besse et al., 2017). Till now, only two mutations in *SEC61B* (p.M1V and p. N36KfsX42) have been reported to cause PCLD. The patients carrying *SEC61B* mutation present moderate-to-severe PCLD without PKD (Besse et al., 2017).

#### ALG9

In 2019, *ALG9* was also discovered as a novel candidate gene in PCLD. This gene encodes α-1,2-mannosyltransferase that catalyzes the addition of mannose to the N-glycan precursor in ER (Besse et al., 2019) (Figure 1). Similar to *ALG8*, loss of function in *ALG9* also causes congenital disorder of glycosylation (CDG) (Höck et al., 2015; Besse et al., 2019), indicating an essential role of *ALG9* in the quality control of glycoprotein. In the study considering *ALG9* and PCLD, mutations p.W227X and p.R370K were found in *ALG9*. However, the two PCLD patients present moderate-to-severe PKD. To investigate whether ALG9 could cause PCLD, they genetically engineered *ALG9* knock-out cell lines using CRISPR-Cas9 and observed a reduced manner of cell surface-expressed mature PKD1 with an altered N-glycosylation pattern (Besse et al., 2019).

## Discussion

Because the SEC61 translocon complex is critical for the Ca^2+^ channel (Lang et al., 2017), PRKCSH is also shown to directly bind Ca^2+^ by the EF-hand domain and control the channel protein TRPV5 (Gkika et al., 2004). Wnt signaling could also play a role in Ca^2+^ pathway activation (Kühl et al., 2000). The Ca^2+^ pathway might also play a role in the cytogenesis of PCLD. Lower cytoplasmic Ca^2+^ level and ER Ca^2+^ stores were also found in cystic cells (Spirli et al., 2017), indirectly indicating a possible role of Ca^2+^ in PCLD. Moreover, decreased Ca^2+^ level is reported to cause elevation of cAMP and cAMP-dependent Ras/Raf/ERK pathway (Spirli et al., 2017). Increased cAMP can result in biliary cell proliferation and decreased apoptosis, which is also thought to be critical for cytogenesis in PCLD (Drenth et al., 2010).

Besides, except for *LRP5*, all candidate genes of PCLD are ER located and play a role in glycosylation, quality control, and translocation of glycoprotein across the ER (Cornec-Le Gall et al., 2018). Their roles in the glycosylation process are shown in Figure 1. Disordered glycosylation results in their retention in ER (Ushioda et al., 2013) and decreased protein activity, for example, protein PKD1. The bile duct also seems to have a lower tolerance for the PKD1 activity (Besse et al., 2017). This might explain the reason for the cytogenesis in the liver.

Therefore, not only genes involved in glycosylation and translocation of protein across ER but also genes involved in the Ca^2+^ pathway might be considered possible candidate genes in PCLD. Below, we enumerate some promising candidate genes in PCLD.

### *SEC61A1*, *SEC61A2*, *SEC61G*, and *SEC62*

*SEC61A1*, *SEC61A2*, *SEC61G*, and *SEC62* all encode the components of the SEC61 translocon complex. As mentioned before, this complex is critical in the translocation and quality control of glycoproteins. Because mutations in *SEC61B* and *SEC63*, which also encode the protein comprising SEC61 translocon, can result in disruption of the complex and cause PCLD. Other genes involved in the SEC61 complex might also play a role in PCLD.

### ALG6

The same as *ALG8*, *ALG6* also encodes a α-1,3-glucosyltransferase, ALG6 and ALG8 belongs to the same glucosyltransferase family, GT57 (Albuquerque-Wendt et al., 2019). However, ALG6 catalyzes the addition of the first glucose residue to the lipid-linked oligosaccharide chain of the N-glycan precursor, while ALG8 catalyzes the addition of the second glucose (Sun et al., 2005). Because the deficiency in ALG6 is reported the same as ALG8 and ALG9 causing CDG, it reveals an essential role of ALG6 in the quality control of glycoprotein.

### LRP6

*LRP6* and *LRP5* shared 71% of identity in the amino acid level (Brown et al., 1998). They are always co-expressed at the embryonic stage (Pinson et al., 2000) and function together as the co-receptor of Fzd and mediate the Wnt signaling. Since the deficiency in LRP5 resulting in reduced Wnt signaling could be a pathogenic reason for PCLD, deficiency in LRP6 might also be related to PCLD.

## Conclusion

PCLD consists of a heterogeneous group of disorders with different genetic mutations and disease presentations. Glycosylation, translocation, and quality control of protein in ER play a central role in the diseases. However, the established candidate genes can only explain less than half of the cases. More candidate genes of diseases should be explored and investigated in the future.

## Supplementary Material

Supplementary Table S1 The precise information of all reported mutations in *PRKCSH* and *SEC63* gene including chromosomal location and cohort size of the studies.
